# Supplementary material for: Mirror-induced reflection in the frequency domain
Source: Nat Commun. 2022 Oct 22;13:6293. doi: 10.1038/s41467-022-33529-w (PMC9588073; doi:10.1038/s41467-022-33529-w)
Supplement: Supplementary file 2 — Supplementary materials [file 41467_2022_33529_MOESM2_ESM.pdf]

Supplementary materials
Mirror-induced reflection in the frequency domain

Yaowen Hu[1,2,*,†], Mengjie Yu[1,3,*], Neil Sinclair[1,4], Di Zhu[1], Rebecca Cheng[1], Cheng Wang[5] and Marko Lončar[1,†]

[1]*John A. Paulson School of Engineering and Applied Sciences, Harvard University, Cambridge, MA 02138, USA*

[2]*Department of Physics, Harvard University, Cambridge, MA 02138, USA*

[3]*Ming Hsieh Department of Electrical and Computer Engineering, University of Southern California, Los Angeles, CA 90089, USA.*

[4]*Division of Physics, Mathematics and Astronomy, and Alliance for Quantum Technologies (AQT), California Institute of Technology, Pasadena, CA 91125, USA.*

[5]*Department of Electrical Engineering & State Key Laboratory of Terahertz and Millimeter Waves, City University of Hong Kong, Kowloon, Hong Kong, China*

[*]*These authors contributed equally*

[†]*Correspondence to: loncar@seas.harvard.edu; yaowenhu@fas.harvard.edu*

Table S1 | Parameters of the systems

Parameters	Simulation in Fig. 2	Polarization mirror in Fig. 3	Coupled-resonator mirror in Fig. 4	Outlook
Waveguide-ring coupling $\boldsymbol{\kappa_e}$ (MHz)	48	20	39	2
Intrinsic loss rate $\boldsymbol{\kappa_i}$ (MHz)	96	85	169	19
Resonance linewidth $\boldsymbol{\kappa}$ (MHz)	144	105	207	21
Intrinsic $\boldsymbol{Q}$	2.0×10^6	2.7×10^6	1.1×10^6	10×10^6
Loaded $\boldsymbol{Q}$	1.3×10^6	2.2×10^6	0.89×10^6	9.2×10^6
Coupling strength $\boldsymbol{\Omega}$ (GHz)	6	6	6	6
Parameter $\boldsymbol{u = \kappa/\Omega}$	2.4×10^{-2}	1.8×10^{-2}	3.5×10^{-2}	0.35×10^{-2}
Propagation loss $\boldsymbol{L}$ (dB/lattice)	0.1	0.076	0.15	0.015
Pump wavelength (nm)	1550	1309	1628	1550
Mirror coupling strength $\boldsymbol{\mu}$ (GHz)	2	0.86	6.8	5
Free spectral range FSR (GHz)	10	10.5	10.5	10
Parameter **G**	772	268	4296	250627
Reflectivity	0.994	0.94	0.999914	0.999997
Power cut off, simulation (dB)	25.9	15.2	44	58.9
Power cut off, experiment (dB)	N/A	16	>30 (limited by noise floor)	N/A

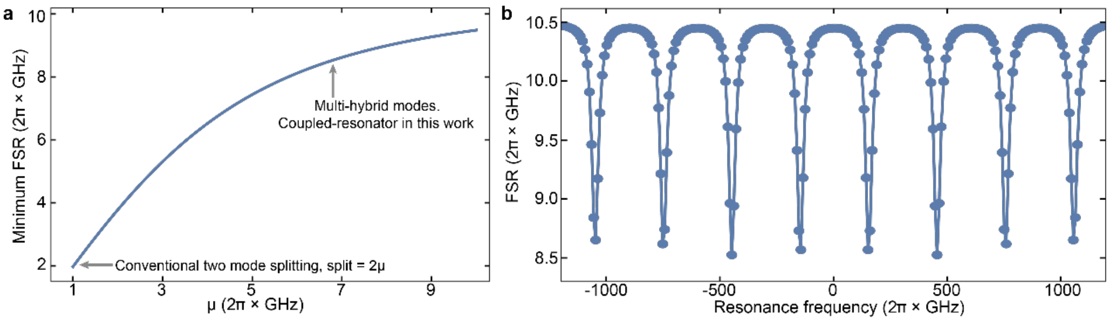

Fig. S1. Relationship between coupling strength and the minimum FSR in the region of gradually reduced FSR. a, Simulated minimum FSR when varying the coupling μ .The phenomenon of gradually reduced FSR happens when the coupling μ starts to become comparable with the FSR of the cavity 1. For example, when the coupling μ is small, it can only couple degenerate modes therefore provide a conventional two mode splitting with a splitting equal to 2μ. When the coupling μ becomes stronger, such that 2μ is larger than the FSR, dispersive coupling starts to contribute, and multi-hybrid modes are formed. We extract the coupling strength μ in our coupled system based on simulation and obtained $\mu = 6.8$ GHz, which satisfies $2\mu >$ FSR. When the coupling μ becomes extremely large, the minimum FSR will reach its original FSR (10.5 GHz in our simulation here). **b,** Simulated FSR as a function of resonance frequency (Fig. 4d) for the coupling extracted from our system ($\mu = 6.8$ GHz).
